# Boceprevir (BOC) and Telaprevir (TPV) therapeutic drug monitoring in HCV and HIV-HCV infected patients treated with triple therapy Ribavirine/Peg-interferon/Boceprevir or Telaprevir: impact of the antiretroviral (ARV) treatment

**DOI:** 10.1186/1471-2334-14-S2-P83

**Published:** 2014-05-23

**Authors:** AS Chantry, M Tching-Sin, C Dhiver, T Allegre, JM Ruiz, A Assi, D Botta-Fridlund, P Halfon, O Faucher-Zaegel, C Solas

**Affiliations:** 1 Timone Hospital, Pharmacokinetics and toxicology department, Marseille, France; 2 AP-HM Conception Hospital, Infectious Diseases department, Marseille, France; 3 Pays d’Aix Hospital, Hematology and Internal Medicine Department, Aix-en-Provence, France; 4 AP-HM, Hôpitaux Sud, Medicine in prison department, Marseille, France; 5 Sainte Musse Hospital, Infectiology department, Toulon, France; 6 AP-HM Conception Hospital, Digestive surgery department, Marseille, France; 7 European Hospital, Marseille, France; 8 Alphabio Laboratory, Marseille, France; 9 AP-HM Sainte-Marguerite Hospital, Immuno-Hematology department, Marseille, France; 10 University of Aix-Marseille, INSERM UMR 911 - CRO2, Faculty of Pharmacy, Marseille, France

## Introduction

BOC and TPV are potent NS3/4A protease inhibitors for the treatment of chronic hepatitis C (HCV) genotype 1 infection. BOC and TPV are both substrates and strong inhibitors of the CYP3A, therefore presenting a wide interindividual pharmacokinetic variability and multiple drug interactions especially with ARV such as lopinavir/r, darunavir/r or efavirenz, thus restricting options for concomitant ARV therapy. We evaluated plasma concentrations of coinfected and monoinfected patients treated with BOC and TPV and the PK data of patients treated with non recommended ARV.

## Method

Data from patients whose BOC and TPV trough concentration had been assessed during treatment were retrospectively analyzed. Plasma concentrations were determined using a LC-MS/MS method. Mann-Whitney U test was used for statistics (PASW Statistics 17).

## Results

Overall, 58 patients were included (84% male, median age: 51 years (34-70)), treated with BOC (25) or TPV (33). Thirty-two (55%) patients are coinfected (14 BOC, 18 TPV) and 26 (45%) are monoinfected (11 BOC, 15 TPV). Median (range, CV) TPV and BOC trough concentrations were respectively, 1928 ng/mL (92-3204, 47%) and 111 ng/mL (33-903, 112%) in coinfected patients versus 2787 ng/mL (252-5551, 54%) and 153 ng/mL (25-2658, 150%) in monoinfected patients, which is statistically different only for TPV (p<0.05). Six patients received non recommended ARV: 4 were treated with darunavir/r (2 BOC, 2 TPV), 1 with efavirenz and BOC and 1 with lopinavir/r and TPV. Median (range) TPV and BOC concentrations were respectively, 1967 ng/mL (580-3204) and 103 ng/ml (33-903) with recommended ARV versus 1304 ng/mL (92-2565) and 146 ng/ml (65-304) with non recommended ARV.

## Conclusion

This study highlights a strong interindividual variability in BOC and TPV trough concentrations. Lower concentrations were observed in coinfected patients but remaining within the expected range, which may be explained by drug interactions with some ARV. Hence, therapeutic drug monitoring is useful to manage these interactions and evaluate the risk-benefit balance of using non recommended ARV in coinfected patients with advanced hepatic disease.

